# Lesion Size Index in Maximum Voltage-Guided Cavotricuspid Ablation for Atrial Flutter

**DOI:** 10.19102/icrm.2017.080603

**Published:** 2017-06-15

**Authors:** Usama Boles, Enes E. Gul, Noel Fitzpatrick, Andres Enriquez, John Conroy, Arian Ghassemian, Santhosh David, Adrian Baranchuk, Christopher Simpson, Damian Redfearn, Benedict Glover, Hoshiar Abdollah, Kevin Michael

**Affiliations:** ^1^Division of Cardiology, Heart Rhythm Service, Queen’s University, Kingston, Ontario, Canada; ^2^Department of Cardiology, Midland Regional Hospital and St. James Hospital, Trinity College, Dublin, Ireland; ^3^Department of Cardiology, Letterkenny University Hospital, Donegal, Ireland

**Keywords:** Ablation, atrial flutter, contact force, lesion size index, maximum voltage-guided

## Abstract

The application of optimum contact force (CF) can be used to improve ablation procedure success and safety. The lesion size index (LSI) is a novel dimensionless contact force parameter that allows for an accurate estimation of lesion volume in real time by integrating contact force (grams), duration (seconds) and power (watts). The aim was to correlate LSI values with current contact force parameters to achieve successful and safe bidirectional block of the cavotricuspid isthmus (CTI) using a maximum voltage-guided (MVG) ablation strategy. Fifteen consecutive patients (age 69 ± 7.9 years, nine males) with symptomatic atrial flutter (AFL) were evaluated and compared with 23 control (age 66.3 ± 10.4 years, 16 males) non-contact force-guided ablation cases. Irrigated-tip force-sensing ablation catheters (TactiCath Quartz™, St. Jude Medical, St. Paul, MN, USA) were used in the CF group to achieve the primary endpoint of complete bidirectional block of the isthmus. In the CF group, a total of 233 radiofrequency (RF) applications were examined. A mean LSI of 6.4 ±1.0 correlated with a force-time integral (FTI) of 581.2 ±230.9 g/s and an average CF of 13.9 ±4.9 g concurrently. Intraprocedural, fluoroscopy time and RF time demonstrated lower trends in the CF group, but no significance with respect to these trends was observed. The secondary endpoint of no reconnection within 20 min after the procedure was equally attained in both groups, and, likewise, the level of safety was comparable. An LSI value of >5 represents a new effective parameter in MVG ablation for the cavotricuspid region that demonstrates a safe profile. Guidance of CTI ablation using LSI and other contact force parameters of CF 13.9 ±4.9 g and FTI 581.2 ±230.9 g/s demonstrated highly effective and safe outcomes.

## Introduction

Radiofrequency (RF) ablation is a first-line therapy in atrial flutter and carries a superior success rate as compared with that of antiarrhythmic drug use.^1^ The use of optimum contact force (CF) during catheter ablation is associated with increased transmural lesion volumes. CF sensing technology has provided additional ability to assess the level of applied force, enabling safe catheter manipulation and optimal clinical outcomes.^2^ Real-time assessment of CF is important given that higher CF may be potentially hazardous if the median forces exceed 60 g in patients with atrial fibrillation (AF).^3^

The contact force–time integral (FTI) has been employed as an endpoint to inform operators regarding successful lesion creation within the left atria. The FTI reflects the duration (in seconds) of the average contact force (in grams) applied during RF application, with lesion size correlating linearly with measured FTI (grams/ seconds).^4^ A cut-off of 400 g/s or more for FTI has been associated with a higher success rate and lower recurrence of pulmonary vein reconnection compared with an FTI of lower than 400 g/s. Hence, an FTI ≥400 g/s has been suggested as a surrogate end-point to predict the creation of transmural lesions in RF.^5,6^

Booth et al. recently highlighted the new real-time CF parameter of lesion size index (LSI). This novel parameter incorporates RF power, ablation duration, and CF in a dimensionless ratio independent of traditional parameters. Clinically, LSI correlates with satisfactory short- to medium-term results.^7^ However, this novel CF marker has not yet been fully investigated in cavotricuspid isthmus (CTI) ablation.

We elected to investigate the use of LSI during maximum voltage-guided (MVG) ablation of typical atrial flutter (AFL), which is now accepted as a standard ablation strategy in many centers. It aims to concentrate the creation of ablation lesions on the muscle bundles responsible for transisthmus conduction. This method has the advantages of requiring significantly less RF ablation time and procedure time, as it does not necessitate the completion of the CTI line anatomically, as is done in the traditional “drag” technique.^8,9^

Modern RF CTI ablation techniques for typical AFL have already been shown to be safe and effective.^1^ However, we speculate that the utilization of real-time operative parameters incorporating power, CF and time can still offer incremental benefits in terms of outcomes, safety and resource utilization. LSI is one such parameter; in this consecutive case series with controls, we consider the acute success rates, procedure time, fluoroscopy time and RF time. We further speculate that an LSI threshold of 5 may represent an appropriate intraoperative surrogate goal for the safe formation of transmural ablation lesions to achieve successful acute bidirectional block.

## Methods

This was a retrospective single-center study that enrolled 15 consecutive patients who underwent CTI ablation for typical AFL using contact force-enabled catheters, and 23 patient controls who underwent ablation without the use of contact force-enabled catheters. All procedures were conducted under conscious sedation using midazolam and fentanyl boluses intravenously, and local anesthesia using lidocaine 1%, and were performed by two expert operators who carry out a minimum of 100 ablation procedures per year. The study protocol was reviewed and approved by the institutional ethics committee of Kingston General Hospital and Queen’s University in Ontario, Canada, and conformed to the ethical principles outlined by the Declaration of Helsinki.

### Patient demographics

The controls for this study were randomly selected using age- and gender matching from patients admitted to Kingston General Hospital for catheter ablation for typical AFL between January 2014 and December 2015. All control subjects met the selection criteria, and were enrolled in consecutive fashion.

We included patients > 18 years old with documented paroxysmal or persistent AFL in this study, and excluded those who (1) had undergone a previous ablation procedure for AFL; (2) any non-CTI-dependent or atypical AFL, (3) concomitant atrial fibrillation (AF); and/or (4) if they were unwilling to participate in the study.

Patient electronic charts were surveyed for demographics, comorbid conditions, history of cardiac disease, CHADS2 and CHAD2S2-VASc scores, and echocardiographic parameters prior to ablation, including left atrium diameter and left ventricular ejection fraction.

### Catheter set-up

Standard intracardiac catheters were introduced through the patients’ right femoral veins as appropriate for the procedure as follows: (1) first, Decapolar CSL™ catheters (St. Jude Medical, St. Paul, MN, USA) were presented. Next, (2) a 20-pole catheter was positioned along either the right atrial free wall or the tricuspid annulus, and (3) a mapping and ablation catheter (either TactiCath Quartz™, St. Jude Medical, St. Paul, MN, USA; or CoolFlex™, St. Jude Medical, St. Paul, MN, USA) was delivered either through a 9F femoral sheath (St. Jude Medical, Minneapolis, MN, USA) or (4) an Agilis™ steerable sheath (St. Jude Medical, Minneapolis, MN, USA), which was used in some cases for better stability or longer reach to the tricuspid valve annulus. (5) Finally, a quadripolar catheter (Supreme™, St. Jude Medical, St. Paul, MN, USA) was placed at the right ventricular (RV) apex.

### Contact force and ablation procedures

Prior to mapping, the contact force-enabled catheter (TactiCath Quartz™, St. Jude Medical, St. Paul, MN, USA) was calibrated either outside the body or while floating freely in the mid-right atrium, to set the baseline value of contact force at zero grams. Mapping for maximum voltage electrograms (MVEs) was performed first, prior to RF ablation. A 4-mm CoolFlex™ irrigated catheter (St. Jude Medical, St. Paul, MN, USA) was used for ablation in the control group. Power delivery of RF was adjusted to 35 W with catheter irrigation set at 25 ml/min, with 0.9% NaCl.

The MVG RF was applied in both groups until complete bidirectional block was achieved. For each application of RF energy, the following data were extracted: application duration, average CF, FTI, LSI, and the number of RF lesions created. Information on the secondary endpoint of no reconnection after 20 min was also recorded, and any reconnections that occurred were noted.

Procedural duration, fluoroscopy duration, radiofrequency time, and the period of time after the last RF lesion were recorded. The MVE in millivolts (mV) was identified as a target for initial RF application. AFL cycle length (in milliseconds; mm) was measured in those patients who were demonstrating AFL at the time of presentation to the clinic.

### Statistical analysis

Data were analyzed using SPSS software version 21.0 (IBM Co., Armonk, NY, USA) and are presented as mean ± standard deviation (SD) and median (interquartile range (IQR)) values. The distribution of the variables was analyzed with the Kolmogorov–Smirnov test. Differences between the two groups were tested using independent Student’s t-tests for normally distributed variables, and the Mann–Whitney U test for non-parametrically distributed variables. Differences between categorical variables were analyzed using the chi-squared test. A p-value of less than 0.05 was considered to be statistically significant.

## Results

### Patient demographics

A total of 15 patients were enrolled in this study as part of the CF group, versus 23 individuals in the non-CF group. All patients underwent MVE-guided RF ablation. Patient baseline characteristics are outlined in **Table 1,** with no differences between the study cohorts and controls noted.

### Contact force parameters and procedural details

**Figure 1:**
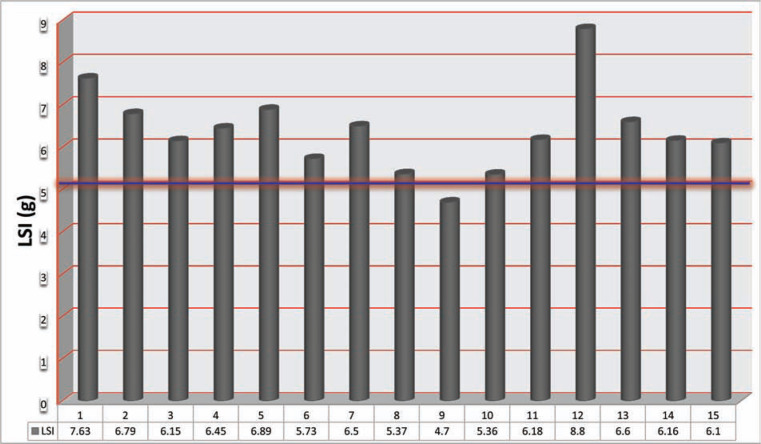
The mean LSI reached in each procedure. A horizontal line here illustrates that the minimum average of LSI > 5 g was predominantly reached.

**Figure 2:**
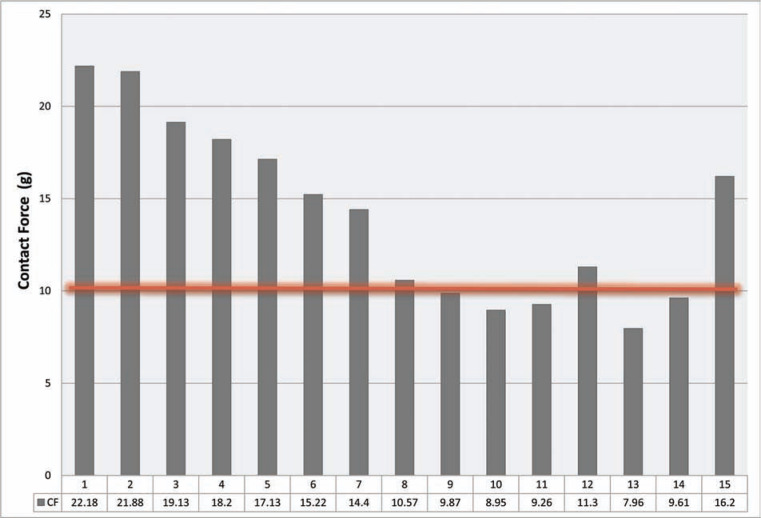
The mean of average CF values reached. A horizontal line illustrates the minimum average CF ≥ 10 g, which was predominantly reached.

**Figure 3:**
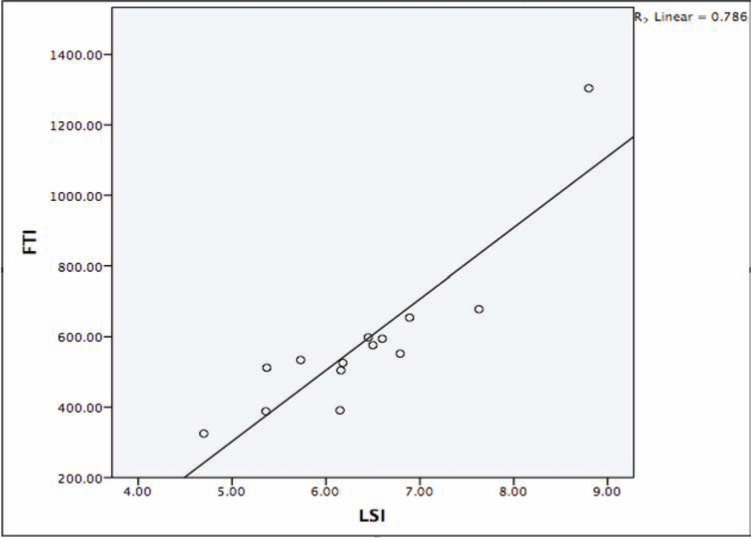
This figure depicts the correlations between LSI and FTI. This correlation was examined in contact force group using TactiCath™ (St. Jude Medical, St. Paul, MN, USA) catheters.

The LSI parameter for lesion depth and volume was 6.4 ± 1.0 **(Figure 1)**. The overall average of CF was 13.9 ± 4.9 g and the average of FTI was 581.2 ± 230.9 g/s **(Table 2** and **Figure 2)**. The results of both LSI and FTI are well correlated (r = 0.8 and p < 0.001) **(Figure 3)**. A trend of lower procedure time, fluoroscopy time, and RF time was observed in the CF group **(Table 3)**. A successful secondary endpoint of no reconnection after 20 min was achieved in all patients, with no complications **(Table 3)**.

### Intraoperative details

**Table 3** presents intraprocedural details for both groups. In the CF group, trends of lower RF time (p= 0.6), number of ablation lesions, fluoroscopy time, and, subsequently, procedure time, were noted.

### AFL at presentation

Eleven patients in the CF group and 12 patients in the non-CF group had AFL as initial rhythm at the beginning of their respective procedures. AFL cycle length, the time to terminate AFL, the number of lesions required, and MVE are outlined in **Table 4.** It is notable that there was a trend in the CF group that led to a lower number of required RF lesions and, subsequently, a shorter time to termination of AFL.

## Discussion

Although AFL ablation is already a highly successful and safe procedure associated with low recurrence rates,^1^ a number of developed technological and methodological innovations have offered possible incremental benefits in terms of efficiency without sacrificing safety or effectiveness.

Redfearn et al. described an ablation strategy that targets MVE in CTI ablation (ie, critical conducting bundles) that has demonstrated similar success rates to the traditional anatomical CTI line formation (the so-called “drag” technique), but which necessitates fewer ablation lesions and lower radiation exposure times.^8,9^ In our cohorts, the number of lesions required to achieve bidirectional block was 8.3 ± 7.6 in the CF group and 13.2 ± 9 in the control (non-CF) group.

The EFFICAS I and II studied identified optimal contact force parameters resulting in more durable pulmonary vein isolation (PVI) in catheter ablation of paroxysmal AF (PAF). These parameters were associated with a lower incidence of gap formation (ie, they achieved effective lesion formation) and, hence, a lower recurrence rate. The recommendations of CF parameters in PVI procedures include a target average CF value of 10–30 g and a minimum FTI value of 400 g for each RF lesion.^5,14,15^ Another landmark study, TOCCASTAR by Reddy et al., confirmed the importance of using CF-guided strategies in meeting primary safety and effectiveness endpoints in PVI ablation procedures.^16^ In results published by Neuzil et al.,^10^ evidence has been offered that supports the concept that an LSI of greater than 5 predicts reconnection risk in PVI ablation. Although the above studies relate to PVI/PAF ablation, it is reasonable to use these results as a guide at least in qualitative, if not quantitative terms, when considering CTI/AFL ablation.

**Figure 4:**
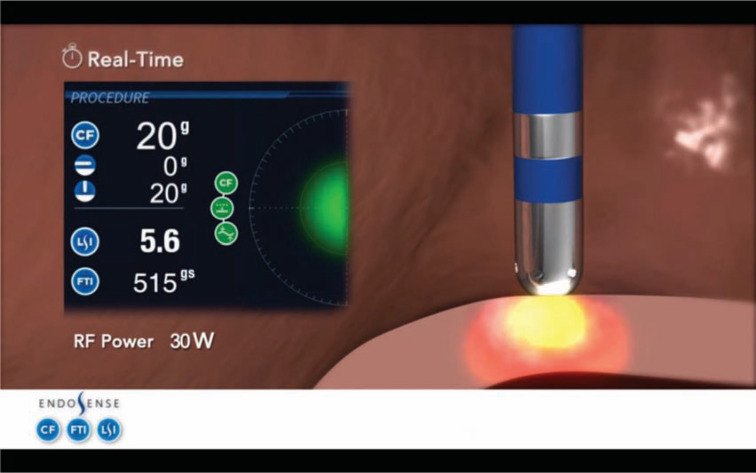
A force-sensing ablation catheter (TactiCath™, St. Jude Medical, St. Paul, MN, USA) showing real-time CF parameters, including CF, LSI and FTI during RF application. Courtesy of Endosense.

Successful RF lesion formation has been shown to be dependent on ablation lesion size and volume, which is associated with good tissue catheter contact and time of RF application.^3,17,18^ Intuitively, successful lesion formation is related to the amount of RF energy transmitted to the prospective lesion site, and is a function of contact force, contact surface area, application time, and application power. Real-time operative parameters to date have considered CF and application time (combined in FTI); however, LSI also incorporates power along with CF and time in a nonlinear fashion to provide a novel dimensionless parameter to guide ablation techniques. Both LSI and FTI are complimentary parameters, yet LSI incorporates the power delivered during RF application **(Figure 4)**.

In this study, we consider the possible impact of using LSI alongside other previously validated real-time operative parameters (CF and FTI) during MVG CTI ablation for typical AFL. In particular, when aiming for an LSI > 5, we show that an LSI of 6.4 ±1.0 reflected a surrogate marker for successful transmural lesion formation, leading to acute bidirectional block of CTI. Although there was no statistically significant difference seen in the intraprocedural data between our study group and the controls, there were trends of less RF application time, fewer ablation lesions, and shorter fluoroscopy and overall procedure times in the LSI/CF-guided patients. Thus, LSI may be reflected as a sufficient parameter once the value of 5 is reached. This LSI value ensures sufficient transmural RF penetration with a safe outcome.^9–13^

### Study limitations

This was a single-center study that involved a small number of patients; therefore, larger randomized studies with power analysis are needed to validate these findings. Additionally, our learning curve with CF catheter use may also influence the results. Furthermore, the aim of this study was to evaluate the effects of LSI and CF parameter use in order to achieve acute procedural success only, and, thus, no follow-up data were included. Future studies incorporating medium- to long-term follow-up with patients could prove to be a valuable resource.

## Conclusions

In MVG cavotricuspid ablation, an LSI >5 (6.4 ±1.0) generated an average CF value of 13.9 ±4.9 g and an FTI of 581.2 ± 230.9 g/s. The use of these parameters led to an acute success rate and safe application similar to that of the standard accepted techniques. Current experience using LSI is early, and more studies are required to validate it.

## Figures and Tables

**Table 1:** Demographic and Clinical Characteristics of the Patients

Variable	MVG With CF (n = 15)	MVG Without CF (n = 23)	p-value
Age	69 ±7.9	66.3 ±10.4	0.6
Male (n, %)	10 (66.6%)	16 (69.6%)	0.8
Ejection fraction (%)	53.5 ±15.9	51.4 ±22	0.1
LA diameter (mm)	40.4 ±6.8	45.3 ±4.9	0.09
LA volume index	34.6 ±11.2	43.6 ±10.3	0.7
Body mass index	31.7 ± 11.1	33.1 ±6.6	0.5
CHADS2	1.3 ± 1	1.6 ± 1.2	0.9
CHADS2-VASC	2.6 **±1**.6	2.5 ±1.6	0.3
Initial rhythm in AFL (n, %)	11 (73.3%)	11 (47. 8%)	0.5
HTN (n, %)	10 (66.6%)	14 (61%)	0.3
Diabetes mellitus	5 (33.3%)	5 (21.7%)	0.6
Ischemic heart disease	5 (33.3%)	7 (30.4%)	0.3
β-blockers	12 (80%)	14 (61%)	0.6
Calcium channel blockers	2 (13%)	4 (17%)	0.2

CF: contact force; n: number; AFL: atrial flutter; HTN: hypertension; LA: left atrium.

**Table 2:** CF Parameters Achieved During the Procedure

Variable	Mean ± SD	Minimum	Maximum
CF average (g)	13.9 ±4.9	7.9	22.2
FTI (g/s)	581.2 ±230.9	324.9	1300.4
LSI	6.4 ±1.0	1.47	8.8

SD: standard deviation; CF: contact force; FTI: force-time integral; LSI: lesion size index.

**Table 3:** Intraprocedural Data Between the Two Groups

Variable	MVG TactiCath™ CF (n = 15)	MVG (Non-CF) (n = 23)	p-value
RF time (min)	5.1 ±2.3	7.6 ±4.8	0.06
Ablation lesions (n)	8.3 ±7.6	13.2 ± 9	0.4
Fluoroscopy time (min)	10.8 ±8.9	12.3 ± 12.1	0.36
Procedure time (min)	87.7 ± 24.2	93.2 ±46.3	0.35
MVE (mV)	1.86 ±1.4	1.55 ±1.4	0.3
Reconnection after 20 min (n, %)	2 (13%)	2 (8.6%)	0.5

CF: contact force; n: number; RF: radiofrequency; MVE: maximum voltage electrogram.

**Table 4:** Subanalysis of the Group of Patients Who Presented with AFL at the Start of the Procedure

Variable	MVG TactiCath™ CF (n = 11)	MVG (Non-CF) (n = 12)	p-value
Number (%)	11 (73.3%)	12 (52.2%)	0.3
AFI CL (mean ms±SD)	246.8 ±31.7	266.2 ±27.5	0.1
Time to terminate AFL (min)	28.5 ±28.1	29.4 ± 24.8	0.9
Number of lesions needed to terminate AFL	8.3 ±7.6	13.2 ± 9	0.08
MVE (mV)	2.1 ±1.5	1.4 ± 1.5	0.19

CF: contact force; n: number; AFL: atrial flutter; CL: cycle length; MVE: maximum voltage electrogram.
